# Effects of Qingluo Tongbi Decoction on Gut Flora of Rats with Adjuvant-Induced Arthritis and the Underlying Mechanism

**DOI:** 10.1155/2019/6308021

**Published:** 2019-08-18

**Authors:** Yan Huang, Meifeng Li, Lingling Zhou, Deguo Xu, Feiya Qian, Junfeng Zhang, Xueping Zhou

**Affiliations:** ^1^School of Medicine and Life Sciences, Nanjing University of Chinese Medicine, Nanjing 210023, China; ^2^College of Pharmacy, Nanjing University of Chinese Medicine, Nanjing 210023, China; ^3^First Clinical Medical College, Nanjing University of Chinese Medicine, Nanjing 210023, China

## Abstract

Rheumatoid arthritis (RA) is a common chronic systemic autoimmune disease. Recent studies show that gut flora plays an important role in regulating the systemic immune response, and gut dysbacteria are linked with systemic chronic inflammation in the development of RA. Our previous results found that Qingluo Tongbi decoction (QLT) can treat RA effectively. The present study explored the effect of QLT on gut flora in an adjuvant-induced arthritis (AA) rat model. Thirty rats were divided randomly into three groups: a control group, a model group, and a treatment group (*n* = 10 per group). The rats in the model group were injected with complete Freund's adjuvant (FCA), while the treatment group received FCA combined with QLT treatment. After 27 days, the gut flora was profiled by 16S rRNA gene sequencing. The levels of cadherin-11, IL-17*α*, TLR2, and TLR4 proteins in the synovial tissues were detected by western blotting (WB). The results showed that QLT treatment significantly inhibited raw swelling during the 15–27 d period compared with the model group. QLT treatment reversed the ten altered bacterial genera in the model group, and three families (*Lachnospiraceae, Eubacteriaceae,* and *Leuconostocaceae*) were closely related to QLT treatment based on linear discriminant analysis (LDA). Functional prediction showed seven types of predicted functions were related to the QLT treatment, and WB results showed that QLT treatment reversed the increased expression levels of cadherin-11, IL-17*α*, TLR2, and TLR4 in synovial tissues significantly. The expression levels of cadherin-11, IL-17*α*, and TLR2 correlated negatively with the abundance of *Staphylococcus* and *Candidatus_Saccharimonas*. Therefore, RA development was related to gut dysbiosis, and QLT effectively ameliorated RA with decreased inflammatory responses regulated by the gut flora.

## 1. Introduction

Rheumatoid arthritis (RA) is a common chronic systemic autoimmune disease mainly typified by inflammatory cell infiltration, continuous synovial hyperplasia, and cartilage and bone destruction and is accompanied by joint dysfunction and even disability in severe cases [1]. As an autoimmune disease, RA has high incidence rates in developed countries and especially in low- and middle-income countries. The systemic complications, such as cardiovascular disease, seriously affect the patients' quality of life and place a huge burden on society and families [2]. At present, RA is mainly treated by nonsteroidal anti-inflammatory drugs (NSAIDs), glucocorticoids, biopharmaceutical agents, etc. [3]. These drugs can rapidly relieve and control the symptoms of RA but result in toxic side effects, low response rates, and high costs. In recent years, traditional Chinese medicine (TCM) has been used increasingly to treat RA because of its mild toxic side effects and evident therapeutic effects [4].

According to the TCM clinical reorganization, RA is classified as an “arthralgia syndrome.” Professor Zhou, a national Chinese medicine master, and his colleagues discovered that RA commonly manifested itself clinically as “yin deficiency and collateral heat” based on long-term clinical experience. They managed to treat RA effectively using Qingluo Tongbi decoction (QLT) which nourished yin, cleared heat, alleviated arthralgia, and reopened collaterals, with satisfactory outcomes and mild adverse reactions [5].

In view of the significant effect of the Qingluo Tongbi decoction on RA, a QTL granule was prepared for clinical and animal investigations, which included eight herbs (*Panax notoginseng* (Burk.) F. H. Chen, *Sinomenium acutum*, *Bombyx batryticatus*, *Rehmannia glutinosa* (Gaertn.) Libosch, *Tripterygium wilfordii* Hook. f., *Taxillus chinensis* (DC.) Danser, *Sigesbeckia orientalis* L., and *Trachelospermum jasminoides* (Lindl.) Lem.). Early animal studies in AA rats showed that the QLT granule relieved the decreased bone density of hind limb joints and inhibited the destruction of the cartilage markedly [6] and that the granule also inhibited the expression of several inflammatory cytokines, such as plasma TNF-*α*, TNF-*α*, and IL-1 in synoviocytes [7, 8]. The clinical experiment found that the QLT granule inhibited the abnormal proliferation of synovial fibroblasts and the expression of receptor activator of nuclear factor-*κ*B ligand (RANKL) in RA patients [9].

In order to reveal the underlying molecular mechanism of QTL in effectively treating RA, the decoration was simplified to five herbs (*Panax notoginseng* (Burk.) F. H. Chen, *Sinomenium acutum*, *Bombyx batryticatus*, *Rehmannia glutinosa* (Gaertn.) Libosch, and *Tripterygium wilfordii* Hook. f.) which exhibited therapeutic effects on the RA model induced by collagen II in DBA/1 mice [10]. In order to investigate the correlation between synovial inflammation and gut flora composition, the present study utilized the rat model with adjuvant-induced arthritis (AA), and the dosage of the decoction in the present study was converted according to body surface area.

As we all know, gut flora had recently been reported to participate in regulating systemic immune responses, sometimes leading to systemic chronic inflammation [11]. Additionally, the oral flora and gut flora of RA patients are significantly different from those of normal subjects [12], and reductions in gut flora diversity were related to the RA course and autoantibody levels in RA patients [13]. For instance, Maeda and Takeda found that the genus *Prevotella* in the intestinal tract suppressed the progression of arthritis, which was associated with the Th17 response [14]. Taken together, these findings indicate that the gut flora may be involved in the pathogenesis of RA.

Our previous study established an AA rat model to verify that QLT controlled the progression of arthritis by inhibiting the expression of IL-1 and TNF-*α* in plasma and synoviocytes [7] and the activation of tumor necrosis factor receptor-associated factor 6, extracellular signal-regulated protein kinase 1/2, and c-Jun *N*-terminal kinase in osteoclasts, together with osteoclast differentiation and maturation [15]. Based on these promising results, we herein evaluated the effects of QLT on the gut flora of rats with adjuvant-induced arthritis, aiming to provide novel scientific observations to inform the unraveling of the biological mechanism underlying QLT treatment.

## 2. Materials and Methods

### 2.1. Animal Feeding and Drug Preparation

Thirty SPF-grade male adult Sprague Dawley (SD) rats weighing 160–180 g (about 2 months old) were purchased from the Laboratory Animal Center of Zhejiang University (production license: SCXK (Zhejiang Province): 2014-001) and kept at the Laboratory Animal Center of Nanjing University of Chinese Medicine. With free access to standard feed and tap water, they were fed under artificial light for 12 h daily at room temperature (23 ± 2°C) with a relative humidity of 50%–60% for one week.

**Figure 1:**
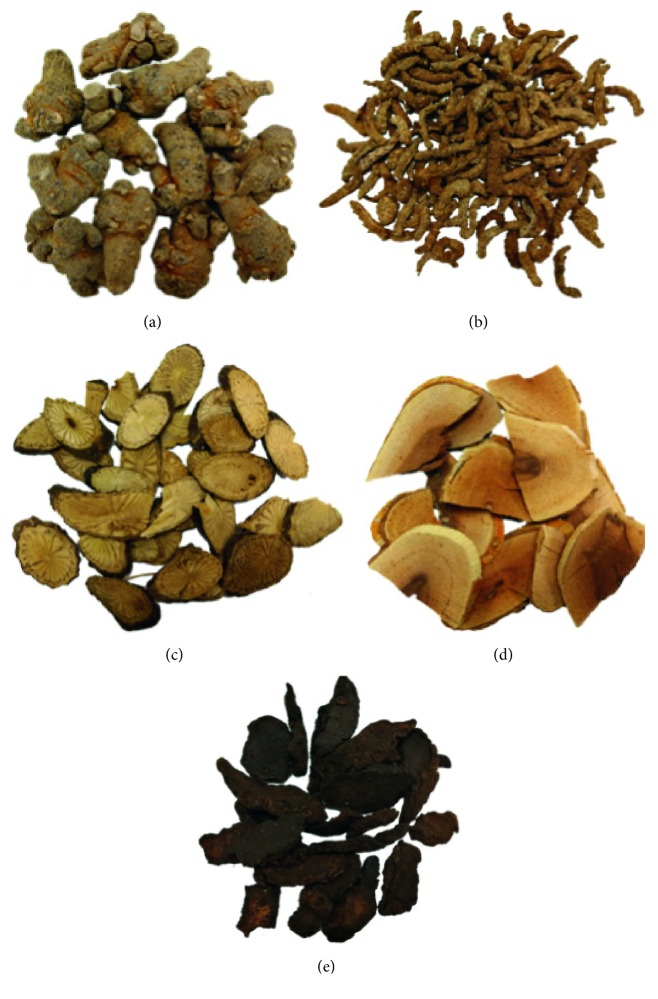
The five herbs in QLT: (a) *Panax notoginseng* (Burk.) F. H. Chen; (b) processed *Bombyx batryticatus*; (c) *Tripterygium wilfordii* Hook. f.; (d) *Sinomenium acutum*; (e) *Rehmannia glutinosa* (Gaertn.) Libosch.

QLT comprised 3 g *Panax notoginseng* (Burk.) F. H. Chen, 15 g *Sinomenium acutum*, 10 g processed *Bombyx batryticatus*, 15 g *Rehmannia glutinosa* (Gaertn.) Libosch, and 5 g *Tripterygium wilfordii* Hook. f. (Figure 1). All the medicinal materials were bought from the Guoyi Clinic of Nanjing University of Chinese Medicine and authenticated by Professor Chen Jianwei of College of Pharmacy, Nanjing University of Chinese Medicine. The medicinal materials were immersed in cold water for 1 h, boiled over intense heat, simmered for 25 min, and decocted twice. The water decoction was manufactured into a freeze-dried powder, and the redissolved solutions were stored at 4°C at a concentration equivalent to 0.432 g/ml crude drug.

Samples of QLT were separated on a Macherey-Nagel Nucleodur Gravity-SB C18 column (250 mm × 4.6 mm, 5 *μ*m), and the mobile phase consisted of acetonitrile (A) and phosphate buffer (B). The mobile phase flow rate was 1.0 ml/min, and the column temperature was controlled at 40°C. A Shimadzu diode array detector was set at 203 nm to detect the constituents of the QLT.

### 2.2. Establishment of the RA Model and Drug Intervention

Thirty rats were divided randomly into three groups: a control group, a model group, and a treatment group (*n* = 10 per group). The AA model was produced as described previously [16]. In brief, the rats in the model and treatment groups were injected intradermally with FCA (liquid paraffin :  lanolin = 3 : 2, 10 mg/ml BCG; total volume: 0.1 ml) into the plantar surface of the left hind paw, while the rats in the control group were injected with the same volume of normal saline. Then, the rats in the treatment group were given 4.32 g QLT crude drug/kg body weight by intragastric administration once daily, while the rats in the control and model groups were given 2 ml distilled water once daily. The swelling of the left hind paw plantar surface and the secondary changes in joints were observed daily until the 27th day. The progression and severity of pathological changes were scored by using the arthritis index: 0 points: normal; 1 point: mild redness or swelling of only regional parts or toes; 2 points: moderate swelling of toe joints, paw plantar surface or ankle joints; 3 points: severe swelling of ankle joints or complete swelling below ankle joints; and 4 points: swelling of the entire paw or severe deformation of joints.

### 2.3. Detection of Gut Flora and Data Processing

Before scarification, two fresh feces pellets were collected and stored at −80°C. The gut flora was detected by Shanghai Majorbio Bio-Pharm Technology Co., Ltd. (China). In brief, total DNA was extracted from the feces with the E.Z.N.A.® Soil DNA extraction kit (Omega Bio-tek, Norcross, GA, USA) and subjected to PCR amplification using 16S rDNA universal primers (338F-ACTCCTACGGGAGGCAGCAG, 806R-GGACTACHVGGGTWTCTAAT). The PCR products were used to prepare a MiSeq PE library for high-throughput sequencing. The raw data were quality-controlled and screened with QIIME software (version 1.17), and the optimized sequences were employed to establish operational taxonomic units (OTUs) at 97% similarity with UPARSE software (version 7.1; http://drive5.com/uparse). Bioinformatics analysis was performed by using online software (http://www.i-sanger.com), including alpha diversity analysis, beta diversity analysis, gut flora composition analysis, linear discriminant analysis effect size (LEfSe), and functional prediction using Kyoto Encyclopedia of Genes and Genomes (KEGG).

### 2.4. Western Blotting

The rats were deprived of food and water for 24 h after the final intragastric administration and sacrificed by dislocation, from which the synovial tissues of the knee joint were collected, immediately snap-frozen in liquid nitrogen for 2 h, and stored at −80°C. Afterwards, proteins were extracted from the tissues to measure the expression levels of cadherin-11, TLR2, TLR4, and IL-17*α* by western blotting. In brief, 70 *μ*g protein was denatured at 95–100°C, cooled on ice for 5 min, loaded for SDS-polyacrylamide gel electrophoresis, and electronically transferred onto an NC membrane using the wet method. Then, the membrane was blocked with 5% skimmed milk for 2 h and incubated with mouse anti-cadherin-11 (1 : 4000; C4283; LifeSpan BioSciences, Inc.), rabbit anti-TLR2 (1 : 4000; ab191458; Abcam), rabbit anti-TLR4 (1 : 4000; C190258; LifeSpan BioSciences, Inc.), rabbit anti-IL-17*α* (1 : 4000; C331074; LifeSpan BioSciences, Inc.), and rabbit anti-*β*-actin (1 : 4000; ab8227; Abcam) by shaking overnight at 4°C. Subsequently, the membrane was washed four times with PBST (10 min each time), incubated with horseradish peroxidase-labeled goat anti-rabbit secondary antibody (1 : 5000; 111-035-003; Jackson ImmunoResearch Laboratories, Inc.) or horseradish peroxidase-labeled goat anti-mouse secondary antibody (1 : 5000; 115-035-003; Jackson ImmunoResearch Laboratories, Inc.) at room temperature for 2 h, and washed with PBST four times (10 min each time). Finally, the membrane was exposed to the X-ray film and photographed using an HP scanner (HP Laser Jet 1536 dnf MFP, USA).

### 2.5. Statistical Analysis

All data were analyzed using SPSS software (version 21.0; SPSS Inc., Chicago, USA). The data were presented as mean ± SD, and the statistical methods applied included ANOVA, Student's *t*-test, nonparametric test (Mann–Whitney *U* test or Kruskal–Wallis *H* test), and Spearman's correlation analysis. The correlated heatmap was presented by R software (https://www.r-project.org/).

## 3. Results

### 3.1. Qualitative Analysis of Bioactive Compounds in QLT

To ensure consistency and reproducibility, the representative chemical compositions of the QLT were determined by high-performance liquid chromatography (HPLC). Six chemical components from the herbal medicine were identified and quantified by HPLC, except the animal medicine (processed *Bombyx batryticatus*) ([Supplementary-material supplementary-material-1]).

### 3.2. Effects of QLT on Adjuvant-Induced Arthritis

**Figure 2:**
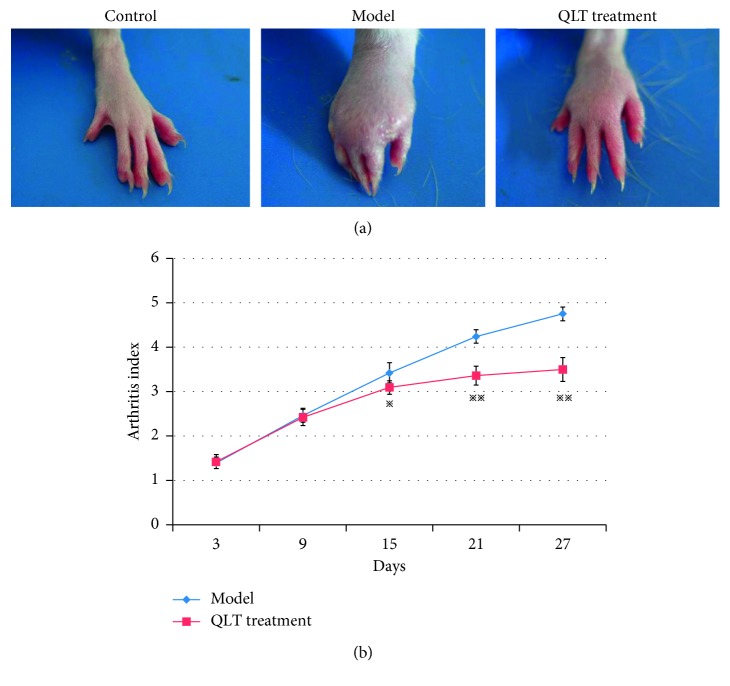
QLT treatment inhibited the paw swelling in the AA rats. (a) Representative photos of paw swelling of each group on the 27th day. (b) Arthritis index of paw swelling during 27 days between the model and the QLT treatment. Two-way ANOVA: ^*∗*^ *P* < 0.05 and ^*∗∗*^ *P* < 0.01, in comparison with the model group; *n* = 10 per group. Data are depicted as mean ± SD.

On the 2nd day after FCA injection, the plantar surface of the left hind paws swelled. After 10 days, the redness and swelling aggravated, the sole thickened, and activity of the rats was clearly restricted. Meanwhile, the right hind paw plantar surface was hardly red or swollen, and appetite decreased slightly. After 20 days, the redness and swelling of the left hind paws of rats in the model group further progressed. In contrast, the rats in the treatment group exhibited improved posture and appetite, as well as mitigated swelling (Figure 2). On the 15th day, the arthritis index of the treatment group was 3.09 ± 0.15, which was significantly lower than that of the model group (3.42 ± 0.23) (*P* < 0.05). On the 21st day, the average arthritis index of rats in the treatment group was significantly lower than that in the model group (*P* < 0.001). Notably, the arthritis index of the model group on the 27th day after challenge significantly exceeded that on the 21st day (*P* < 0.001). However, the arthritis index of the treatment group remained stable (*P* > 0.05). Therefore, QLT started to exert significant therapeutic effects on adjuvant-induced arthritis from the 15th day and prevented paw swelling from the 21th day after challenge.

### 3.3. Effects of QLT on Gut Flora of Rats with Adjuvant-Induced Arthritis

High-throughput sequencing of gut flora of the three experimental groups generated 943790 effective sequences, with an average of 30444.8 ± 3586.1. The dilution curve revealed a saturated sequencing depth, and beta diversity analysis suggested significantly different compositions among the three groups ([Supplementary-material supplementary-material-1]). However, the nonparametric test of alpha diversity indices showed no significant differences among the gut flora compositions of the control, model, and treatment groups (Table 1).

The present study identified 10 phyla, 18 classes, 26 orders, 46 families, 128 genera, 237 species, and 841 OTUs. The community structures of the gut flora are shown in [Supplementary-material supplementary-material-1]. The dominant phyla (relative abundance >1%), in the descending order, were *Firmicutes* (64.7 ± 10.0%), *Bacteroidetes* (29.2 ± 9.8%), and *Proteobacteria* (4.5 ± 2.8%) ([Supplementary-material supplementary-material-1]). In addition, the dominant genera (relative abundance >1%), in the descending order, were *Lachnospiraceae_NK4A136_group* (12.0 ± 5.4%), *Bacteroides* (4.9 ± 4.8%), *[Eubacterium]_coprostanoligenes*_group (3.8 ± 2.7%), *Desulfovibrio* (3.6 ± 2.8%), *Lactobacillus* (3.4 ± 6.4%), *Prevotellaceae_NK3B31_group* (3.4 ± 2.3%), *Ruminococcaceae_UCG-014* (2.9 ± 1.6%), *Ruminiclostridium_9* (2.6 ± 1.1%), *Ruminococcaceae_NK4A214_group* (2.4 ± 1.4%), *Ruminococcaceae_UCG-005* (2.0 ± 3.1%), *Ruminococcus_1* (1.9 ± 1.2%), *Oscillibacter* (1.6 ± 0.7%), *Roseburia* (1.5 ± 1.2%), *Alloprevotella* (1.0 ± 1.5%), [*Eubacterium*]*_xylanophilum*_group (1.0 ± 0.9%), *Ruminiclostridium* (1.0 ± 0.6%), *Ruminococcus_2* (1.0 ± 0.9%), and *Ruminiclostridium_6* (1.0 ± 1.0%) ([Supplementary-material supplementary-material-1]).

**Figure 3:**
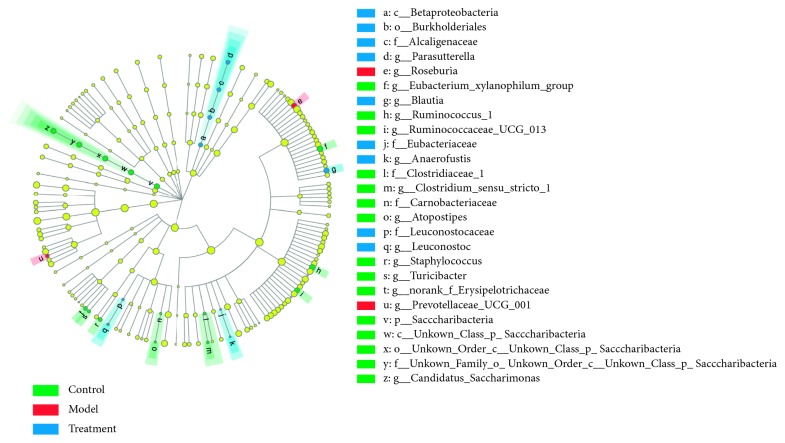
LEfSe analysis of gut flora among the three groups. The symbols before the bacterial names mean the taxonomic level; of them, p_ means phylum, c_ means class, o_ means order, f_ means family, and g_ means genus.

Moreover, LEfSe analysis was conducted to assess the effects of QLT on the gut flora of RA rats from the phylum to the genus levels (Figure 3). The results showed that the flora related to QLT treatment was the genus *Parasutterella*, belonging to the phylum *Proteobacteria*, and also that two genera (*Roseburia* and *Prevotellaceae_UCG_001*) could represent the AA rat's symbolic flora.

**Figure 4:**
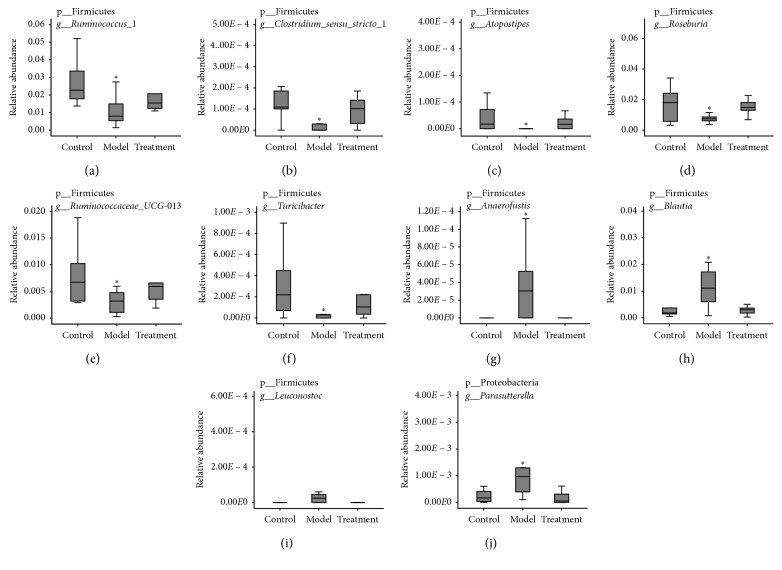
Reversed effects of QLT treatment on the gut flora at the genus level in the AA rats. ^*∗*^ *P* < 0.05, in comparison with the control group.

To assess the effect of QLT treatment in reversing gut flora composition at the genus level in the AA rats, the nonparametric test (Mann–Whitney *U* test) was conducted to comparatively analyze the relative abundance of gut bacterial genera between two arbitrary groups. Ten genera were collected from the total of 128 genera identified because the abundance of each genus was significantly different between the control and model groups and between the model and treatment groups; however, they did not differ significantly between the control and treatment groups. In comparison with the control group, the relative abundance of six genera (*Ruminococcus_*1, *Clostridium_sensu_stricto_1*, *Atopostipes*, *Turicibacter*, *Ruminococcaceae_UCG-013*, and *Roseburia*) significantly decreased (*P* < 0.05) in the model group, while the relative abundance of four genera (*Anaerofustis*, *Blautia*, *Parasutterella*, and *Leuconostoc*) distinctly increased (*P* < 0.05). QLT treatment significantly changed the abundance of genera in AA rats back to their abundance in the control group, and statistical analysis showed that the relative abundance of the above 10 genera in the treatment group was not statistically different from that in the control group (*P* > 0.05) (Figure 4).

### 3.4. Functional Prediction and Validation of Gut Flora

**Figure 5:**
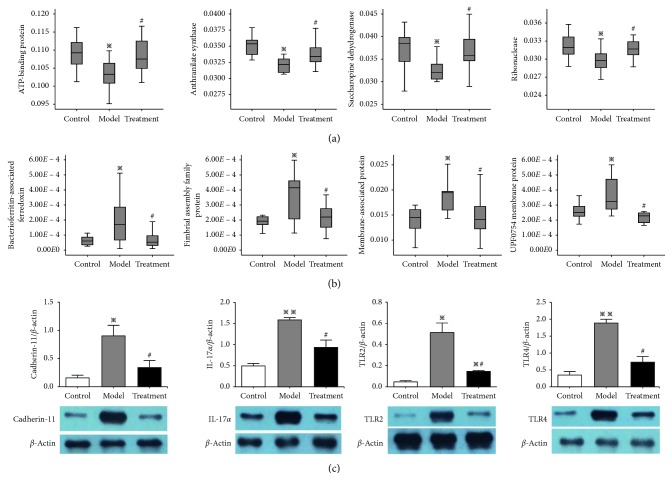
Analysis and verification of QLT treatment-related predictive functions of gut flora in the AA rats. (a) The QLT treatment could decrease four predictive functions. (b) WB analysis of the expressed levels of cadherin-11, IL-17*α*, TLR2, and TLR4 in the synovial tissues was conducted to vivificate the QLT treatment-related predictive functions. (c) Expressed levels were presented as mean ± SD. ^*∗*^ *P* < 0.05 and ^*∗∗*^ *P* < 0.01, compared with the control group. ^#^ *P* < 0.05, compared with the model group.

KEGG was thereafter used for functional prediction to screen the gut flora related to QLT therapy, with the criteria of significant differences among the three groups: significant differences between the control and model groups (*P* < 0.05) and between the model and treatment groups (*P* < 0.05) were observed, but no significant difference between the treatment and control groups (*P* > 0.05) was found. A total of 32 predictive functions were screened. Compared with the control group, the model group had 21 significantly enhanced functions and 11 significantly weakened ones (*P* < 0.05) ([Supplementary-material supplementary-material-1]). According to the pathogenesis of RA, eight functions warranted further research (Figure 5). QLT significantly upregulated ATP-binding protein, anthranilate synthase, saccharopine dehydrogenase, and ribonuclease in the model group (*P* < 0.05) (Figure 5(a)), whereas bacterioferritin-associated ferredoxin, fimbrial assembly family protein, membrane-associated protein, and UPF0754 membrane protein (*P* < 0.05) were downregulated (Figure 5(b)). Furthermore, western blotting showed that compared with the control group, the protein expression levels of cadherin-11, IL-17*α*, TLR2, and TLR4 in the synovial tissues of the model group were significantly upregulated, which, however, were significantly downregulated after QLT administration (*P* < 0.05) to levels similar to those of the control group (*P* > 0.05) (Figure 5(c)).

### 3.5. Correlation between Different Gut Genera and Protein Expression Levels

**Figure 6:**
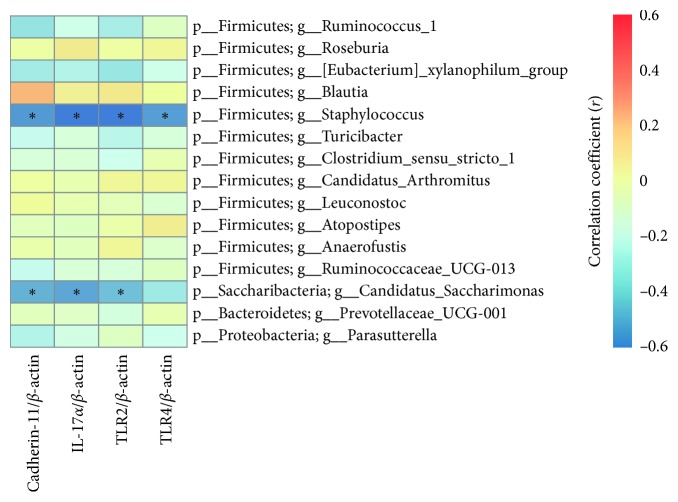
Correlation between different gut genera and protein expression levels. Spearman's correlation analysis was conducted between the relative abundance of 15 different gut genera and the relative expressed levels of verified proteins in the synovial tissues among the three groups. ^*∗*^ *P* < 0.05.

To explore the key gut flora which may trigger the inflammation of synovial tissues, Spearman's correlation analyses were conducted among the 15 known gut genera (shown in Figures 4 and 5) and the expression levels of the four proteins in the synovial tissues. The results revealed that the expression levels of cadherin-11, IL-17*α*, TLR2, and TLR4 in the synovial tissues correlated negatively with *Staphylococcus*, while *Candidatus_Saccharimonas* correlated negatively to a significant degree with the expressed levels of cadherin-11, IL-17*α*, and TLR2 (Figure 6). Both of the two genera were more abundant in the control group (*P* < 0.05).

## 4. Discussion

As a common autoimmune disease, RA can be relieved by combating inflammation and suppressing immunity, and it has been speculated that the inflammatory reaction seen in RA is triggered by complicating factors before the onset of RA, such as gut dysbacteria, opportunistic infection, and unhealthy lifestyle. The human flora participates in regulating physiological metabolism and immune balance, indicating that pathogens that cause RA may exist therein. Jussi et al. found that the abundance of the gut genus *Bifidobacterium* plummeted in patients with early RA or fibromyalgia [17]. In addition, Amdekar et al. proved that *Lactobacillus casei* effectively inhibited collagen-induced arthritis in rats, with comparable results to those of COX-2 inhibitor (indomethacin) [18]. A recent metagenomic study showed that the oral flora and gut flora of Chinese RA patients were significantly different from those of healthy subjects and could be recovered by antirheumatic drugs. Particularly, *Haemophilus* spp. was negatively correlated with serum autoantibody levels, and the relative abundance of *Lactobacillus salivarius* was significantly increased in patients with active RA [12]. Based on functional prediction, the redox environment, transport, and metabolism of iron, together with sulfur, zinc, and arginine were associated with RA. These studies inspired the clarification of mechanisms underlying TCM for prevention and treatment of RA.

Herein, the gut flora of AA rats was changed significantly in control rats and was partly restored by QLT treatment. In comparison with the control group, six genera (*Ruminococcus_1*, *Clostridium_sensu_stricto_1*, *Roseburia*, *Atopostipes*, *Ruminococcaceae_UCG-013*, and *Turicibacter*) significantly decreased in abundance in the model group, while QLT treatment could upregulate the relative abundance of the six genera to the point of having no differences from those in the control group. Furthermore, the relative abundance of four distinct genera (*Anaerofustis*, *Blautia*, *Parasutterella*, and *Leuconostoc*) that was decreased in the model group was also reversed by QLT treatment. Thus, the above ten genera are possible therapeutic markers for QLT. Notably, *Clostridium_sensu_stricto_1* is associated with cellulose digestion and degradation [19, 20], *Roseburia* significantly decreases in patients with Crohn's disease to protect colonic epithelial cells from inflammatory injury [21], and the relative abundance of fecal *Blautia* and *Parasutterella* significantly increases in patients with functional constipation [22]. Hence, effective QLT treatment in AA rats was related to improvement in their intestinal function.

Moreover, QLT managed to reverse thirty-two predictive functions. Of them, four upregulated and four downregulated functions were most crucial in the treatment group compared with the model group in AA rats. The four upregulated functions (ATP-binding protein, anthranilate synthase, saccharopine dehydrogenase, and ribonuclease) were related to the redox state and active inflammation [23, 24], and the four downregulated ones (bacterioferritin-associated ferredoxin, fimbrial assembly family protein, membrane-associated protein, and UPF0754 membrane protein) were associated with gut flora antagonism and mucosal cell integration [25, 26]. The results provide new insight into the molecular biological mechanism underlying the effects of QLT treatment on RA and shed light on the understanding of TCM etiology and pathogenesis in the development of RA.

Among the significantly increased functions, there was particular focus on membrane proteins because of the distinctive effect of QLT in inhibiting paw swelling. Thus, the inflammation-related membrane proteins, TLR2 and TLR4, were selected as they play important roles in the immunity of bacterial infection [27]. Cadherin adhesion molecules are a family of integral membrane proteins that mediate cellular adhesion, which provide cell-to-cell adhesion within tissues, contributing to the maintenance of tissue integrity and architecture. Among them, synovial cadherin-11 determines the behavior of synovial cells in their proinflammatory and destructive tissue response in inflammatory arthritis [28]. Meanwhile, IL-17*α* is well known for its origin in Th17 cells, which has been shown in recent years to play a significant role in the pathogenesis of chronic destructive arthritis [29]. Here, we speculate that the expression of the four above-mentioned molecules in the AA rats' synovium be markedly changed in association with changes in the gut flora.

The results showed that the four molecules, TLR2, TLR4, cadherin-11, and IL-17*α*, were markedly increased in the AA rats' synovium and that QLT treatment can downregulate the expression levels of these proteins. Firstly, cadherin-11 plays key roles in synovial formation and inflammation, as well as in cartilage destruction, which were related to the severity of synovial inflammation [30]. After adhesion, cadherin-11 can promote the mRNA expression of endogenous vascular endothelial growth factor in mouse fibroblasts, angiogenesis, and active synovial inflammation [31]. Secondly, in the development of RA, abnormal proliferation of fibroblast-like synoviocytes and the secretion of inflammatory factors accelerated synovial inflammatory responses, as well as cartilage and bone destruction. The IL-6/SIL-6R complex directly induced RANKL expression in the fibroblast-like synoviocytes of RA patients, where TNF-*α* and IL-17 played essential roles. Additionally, IL-17 exacerbated joint inflammation and destruction by increasing IL-1*β* and IL-6 and also facilitated synovial expression of TLR-2, -4, and -9 in autoimmune arthritis [32]. Finally, TLR2 and TLR4 induced the upregulation of RANKL expression in the fibroblast-like synoviocytes of RA patients [33], damaging the joint surface and stimulating chondrocyte differentiation and eventually leading to bone erosion and injury. In this study, the changes in synovial expression of TLR2 and TLR4 were consistent with those in the above-mentioned literature. Collectively, QLT treatment attenuated the inflammatory activity of synovial tissue, probably in association with IL-17*α* and TLR2- and TLR4-related mucosal immune signaling pathways in AA rats.

The correlation analysis showed that the levels of the four proteins were negatively related to the abundance of *Staphylococcus* and *Candidatus_Saccharimonas*, which had the highest abundance in the control group. Gut *Candidatus_Saccharimonas* was reported to be decreased in rats' acute necrotizing pancreatitis [34], which indicated that *Candidatus_Saccharimonas* plays an important role in maintaining normal intestinal function. Members of the genus *Staphylococcus* have intimate relationships with their hosts, and *Staphylococcus aureus* in particular has been the focus as the pathogenic species in most research including RA patients [35, 36]. Now other *Staphylococcus* species are paid considerable attention because 71 species have been reported to date and little is known about their functions [37, 38]. The results suggest that the development of RA is related to dysbiosis of intestinal flora and especially that the normal gut flora may protect against RA. Thus, more work needs to be done to establish the relationship between gut dysbiosis and RA.

In summary, QLT exerted remarkable therapeutic effects on the inhibition of paw swelling in AA rats, which correlated with the alteration of gut microbiota and with the inhibition of synovial inflammation. The present study paves the way for elucidating the biological mechanism of the role of QLT treatment in RA. Of course, there were two limitations in the present study: (1) a fourth healthy group was not prepared for examining whether QTL exerts an effect on gut microbiota in healthy rats and (2) TCM treatments characteristically have multiple targets; thus, the detailed active constituent and underlying pharmacological mechanism need further study in order to fully understand the effects of QLT therapy on rheumatoid arthritis.

## Figures and Tables

**Table 1:** Influence of QLT treatment on the alpha diversity among the three groups.

	Control (*n* = 10)	Model (*n* = 10)	Treatment (*n* = 10)	*Z* (*P*)^a^	*Z* (*P*)^b^	*Z* (*P*)^c^
Sobs	554.2 ± 22.6	556.7 ± 18.2	539.8 ± 54.3	0.076 (0.940)	0.317 (0.751)	0.423 (0.672)
Shannon	4.645 ± 0.101	4.698 ± 0.130	4.471 ± 0.295	1.134 (0.257)	1.268 (0.205)	1.972 (0.049)
Simpson	0.024 ± 0.004	0.021 ± 0.005	0.031 ± 0.016	1.663 (0.096)	0.423 (0.673)	1.901 (0.057)
Ace	616.3 ± 22.6	618.0 ± 16.9	600.1 ± 62.6	0.000 (1.000)	0.282 (0.778)	0.493 (0.622)
Chao	623.2 ± 24.4	627.7 ± 19.8	609.9 ± 66.1	0.000 (1.000)	0.141 (0.888)	0.352 (0.725)

^a^Control vs model; ^b^control vs treatment; ^c^model vs treatment.

## Data Availability

The data used to support the findings of this study are included within the article.

## Supplementary Materials

Supplementary Materials Figure S1: representative chromatogram of major compounds marked by six main identified compounds originating from the four herbs in QLT. Figure S2: diversity of gut flora. (a) The rarefaction curves showed that the sequencing depth was enough to obtain the total gut flora. (b) Beta diversity analysis presented the significant differences of gut flora among the three groups, and each point represented an individual rat. Figure S3: the community structure of the gut flora in the three groups. (a) The phylum-level community structure of gut flora was presented by heatmap. (b) The relative abundance of top 50 genera in gut flora was presented by heatmap. Table S1: predictive functions related to QLT treatment in AA rats.
