# Supplementary material for: Effects of Qingluo Tongbi Decoction on Gut Flora of Rats with Adjuvant-Induced Arthritis and the Underlying Mechanism
Source: Evid Based Complement Alternat Med. 2019 Aug 18;2019:6308021. doi: 10.1155/2019/6308021 (PMC6721445; doi:10.1155/2019/6308021)
Supplement: Supplementary Materials — Figure S1: representative chromatogram of major compounds marked by six main identified compounds originating from the four herbs in QLT. Figure S2: diversity of gut flora. (a) The rarefaction curves showed that the sequencing depth was enough to obtain the total gut flora. (b) Beta diversity analysis presented the significant differences of gut flora among the three groups, and each point represented an individual rat. Figure S3: the community structure of the gut flora in the three groups. (a) The phylum-level community structure of gut flora was presented by heatmap. (b) The relative abundance of top 50 genera in gut flora was presented by heatmap. Table S1: predictive functions related to QLT treatment in AA rats. [file 6308021.f1.pdf]

Supplement

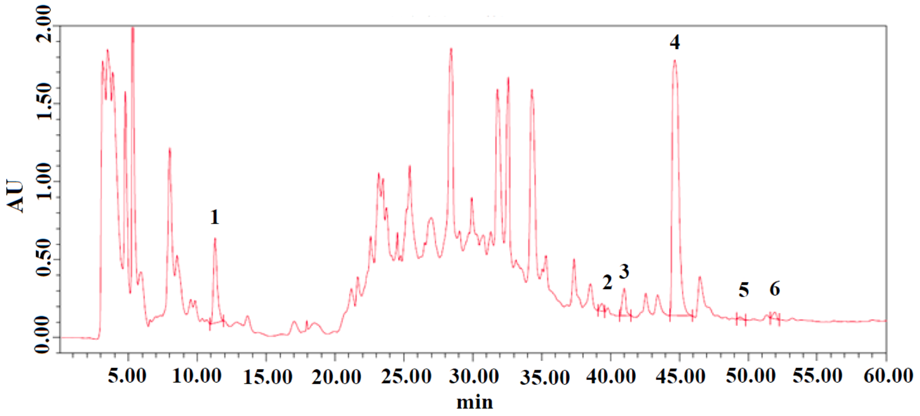

Peak number	Herb	Hallmark of ingredient	Retention time (min)	Percentage of area (%)	Concentration (mg/ml)
1	*Rehmannia glutinosa (Gaertn) Li-bosch*	Catalpol	11.295	14.65	0.7441
2	*Panax notoginseng* (*Burk.*) *F.H.Chen*	Notoginseng saponin R1	39.374	1.13	0.1383
3	*Panax notoginseng* (*Burk.*) *F.H.Chen*	Ginsenoside Rg1	41.005	4.55	0.4931
4	*Sinomenium acutum*	Sinomenine	44.677	78.15	0.5086
5	*Tripterygium wilfordii Hook.f.*	Triptolide	49.398	0.39	0.0136
6	*Panax notoginseng* (*Burk.*) *F.H.Chen*	Ginsenoside Rb1	51.932	1.12	0.1551

Figure S1. Representative chromatogram of major compounds marked by six main identified compounds originated from the four herbs in QLT.

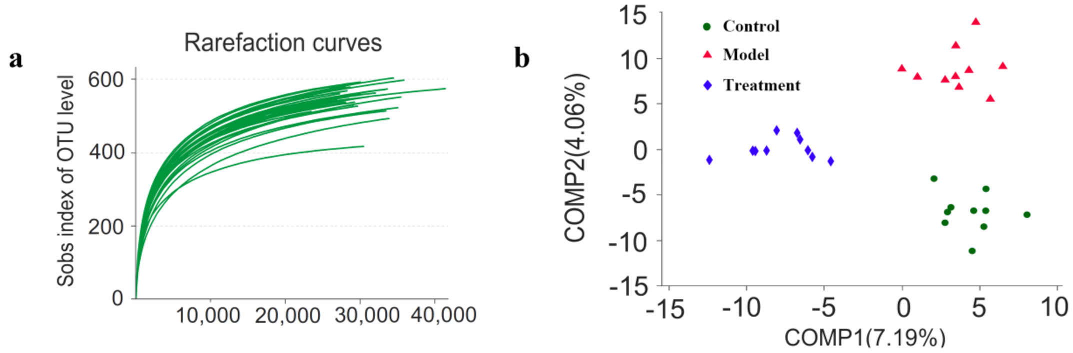

Figure S2. The diversity of gut flora. The rarefaction curves showed that the sequencing depth was enough to obtain the total gut flora (a). Beta diversity analysis presented the significant differences of gut flora among the three groups, and each point represented individual rat (b).

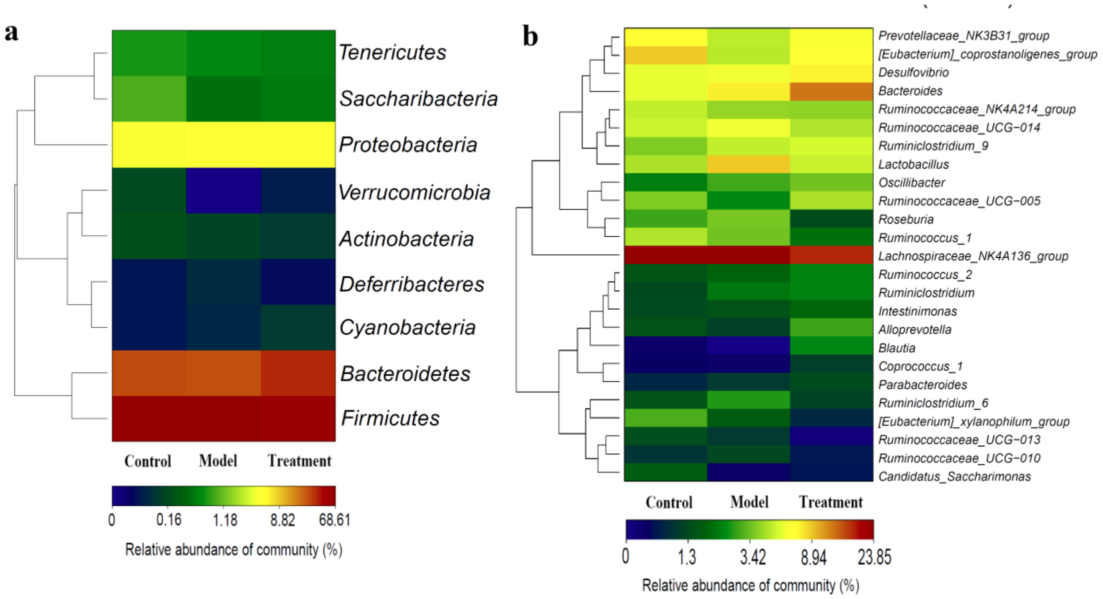

Figure S3. The community structure of the gut flora in the three group. The phylum-level community structure of gut flora was presented by heatmap (a). The relative abundances of top 50 genera in gut flora were presented by heatmap (b).

Table S1. The predictive functions related to QLT treatment in AA rats

ID	**Predictive function**	Control group	Model group	Treatment group	*Z*(*P*)[a]	*Z*(*P*)[b]	*Z*(*P*)[c]	*H*(*P*)[d]
2272	Aminoglycoside	0.66±0.20	0.93±0.40	0.62±0.23	2.117(0.034)	1.972(0.049)	0.141(0.888)	5.601(0.061)
507	anthranilate synthase	3.51±0.18	3.16±0.22	3.40±0.24	3.250(0.001)	2.113(0.035)	1.408(0.159)	11.358(0.003)
484	ATP-binding protein	10.88±0.45	10.35±0.44	10.85±0.47	2.343(0.019)	2.394(0.017)	0.211(0.833)	7.566(0.023)
2379	Bacterioferritin-associated ferredoxin	0.01±0.00	0.02±0.02	0.01±0.01	2.192(0.028)	1.972(0.049)	0.000(1.000)	5.810(0.055)
1557	Bidirectionally degrades single-stranded DNA into large acid-insoluble oligonucleotides, which are then degraded further into small acid-soluble oligonucleotides (By similarity)	2.49±0.33	2.73±0.29	2.43±0.30	2.192(0.028)	2.042(0.041)	0.211(0.833)	6.051(0.049)
1992	Catalyzes the conversion of L-arabinose to L-ribulose (By similarity)	1.40±0.19	1.57±0.13	1.39±0.24	2.192(0.028)	2.253(0.024)	0.070(0.944)	6.647(0.036)
65	Catalyzes the isomerization between 2-isopropylmalate and 3-isopropylmalate, via the formation of 2-isopropylmaleate (By similarity)	4.99±0.13	4.95±0.30	4.98±0.12	2.343(0.019)	2.605(0.009)	0.211(0.833)	8.301(0.016)
735	Catalyzes the NADP-dependent rearrangement and reduction of 1-deoxy-D-xylulose-5-phosphate (DXP) to 2-C-methyl-D-erythritol 4-phosphate (MEP) (By similarity)	4.18±0.21	3.94±0.20	4.17±0.17	2.192(0.028)	2.324(0.02)	0.141(0.888)	6.869(0.032)
1691	CRISPR-associated autoregulator DevR family	0.00±0.00	0.01±0.01	0.00±0.00	2.570(0.010)	2.932(0.003)	0.635(0.526)	10.477(0.005)
2602	cytoplasmic protein	0.01±0.00	0.02±0.02	0.01±0.01	2.192(0.028)	1.972(0.049)	0.000(1.000)	5.810(0.055)
3101	Death-On-Curing Family	0.67±0.20	0.86±0.24	0.62±0.22	2.041(0.041)	1.972(0.049)	0.070(0.944)	5.402(0.067)
4170	DNA alkylation repair enzyme	3.40±0.22	3.16±0.21	3.52±0.47	2.343(0.019)	2.183(0.029)	0.423(0.673)	6.993(0.030)
1001	Facilitates transcription termination by a mechanism that involves Rho binding to the nascent RNA, activation of	3.23±0.21	2.95±0.27	3.24±0.31	2.343(0.019)	1.972(0.049)	0.070(0.944)	6.224(0.045)

	Rho's RNA-dependent ATPase activity, and release of the mRNA from the DNA template (By similarity)							
2628	Fimbrial assembly family protein	0.02±0.01	0.04±0.02	0.02±0.01	2.268(0.023)	1.972(0.049)	0.423(0.673)	6.169(0.046)
3235	Glutathione-dependent formaldehyde-activating Gfa	0.01±0.00	0.02±0.02	0.01±0.01	2.646(0.008)	2.042(0.041)	0.211(0.833)	7.445(0.024)
2055	Isocitrate lyase	0.01±0.00	0.02±0.02	0.01±0.01	1.965(0.049)	2.113(0.035)	0.070(0.944)	5.606(0.061)
406	Key enzyme in heme biosynthesis. Catalyzes the oxidative decarboxylation of propionic acid side chains of rings A and B of coproporphyrinogen III (By similarity)	0.02±0.01	0.03±0.02	0.02±0.01	2.343(0.019)	1.972(0.049)	0.563(0.573)	6.491(0.039)
3386	MazG family	3.16±0.43	2.77±0.30	3.19±0.42	2.041(0.041)	2.183(0.029)	0.070(0.944)	6.007(0.050)
3908	membrane-associated protein	1.49±0.43	1.88±0.32	1.44±0.42	2.343(0.019)	2.394(0.017)	0.070(0.944)	7.547(0.023)
134	N-(5'-phosphoribosyl)anthranilate isomerase	3.75±0.26	3.45±0.18	3.73±0.29	2.721(0.007)	2.817(0.005)	0.211(0.833)	10.338(0.006)
2585	nucleoprotein polynucleotide-associated enzyme	0.01±0.00	0.02±0.02	0.01±0.01	2.192(0.028)	1.972(0.049)	0.000(1.000)	5.810(0.055)
3829	periplasmic	0.01±0.00	0.02±0.01	0.01±0.01	2.343(0.019)	2.113(0.035)	0.141(0.888)	6.645(0.036)
2430	protein involved in catabolism of external DNA	0.02±0.01	0.03±0.02	0.02±0.01	2.041(0.041)	2.113(0.035)	0.282(0.778)	5.841(0.054)
2624	Removes the pyruvyl group from chorismate, with concomitant aromatization of the ring, to provide 4-hydroxybenzoate (4HB) for the ubiquinone pathway (By similarity)	0.01±0.00	0.02±0.02	0.01±0.01	2.192(0.028)	1.972(0.049)	0.000(1.000)	5.810(0.055)
1368	ribonuclease	3.21±0.23	2.94±0.26	3.18±0.22	2.117(0.034)	2.113(0.035)	0.423(0.673)	6.101(0.047)
1583	saccharopine dehydrogenase	3.73±0.44	3.30±0.31	3.70±0.48	2.419(0.016)	2.113(0.035)	0.352(0.725)	6.993(0.030)
2627	sterol-binding domain protein	0.01±0.00	0.02±0.02	0.01±0.01	2.192(0.028)	1.972(0.049)	0.000(1.000)	5.810(0.055)
2410	the catalytic subunit of succinate dehydrogenase (SDH). SDH is involved in complex II of the mitochondrial electron transport chain and is responsible for transferring electrons from succinate to ubiquinone (coenzyme Q). In is unclear whether it participa	0.01±0.01	0.03±0.02	0.02±0.01	2.192(0.028)	1.972(0.049)	0.352(0.725)	5.910(0.052)

1360	transcriptional regulator AsnC family	6.95±0.26	6.61±0.33	7.00±0.38	2.268(0.023)	2.324(0.02)	0.000(1.000)	7.088(0.029)
4223	type IV pilus modification protein PilV	0.04±0.02	0.07±0.03	0.03±0.03	2.419(0.016)	2.676(0.007)	1.549(0.121)	10.007(0.007)
2850	UPF0249 protein	0.77±0.19	0.91±0.18	0.72±0.21	2.041(0.041)	2.042(0.041)	0.493(0.622)	5.703(0.058)
3755	UPF0754 membrane protein	0.03±0.01	0.04±0.01	0.02±0.01	2.041(0.041)	3.098(0.002)	1.620(0.105)	11.054(0.004)

a, model group vs control group; b, treatment group vs model group; c, treatment group vs control group; d, comparison among the three groups.
